# Targeting the immune microenvironment in blastic plasmacytoid dendritic cell neoplasm: a genomic perspective

**DOI:** 10.37349/etat.2026.1002410

**Published:** 2026-10-27

**Authors:** Luigi Cimmino, Martina Lopiano, Valeriia Tsekhovska, Pietro Cimatti, Irma Lapaj, Pier Paolo Piccaluga

**Affiliations:** University of Southampton, UK; N.N. Petrov Research Institute of Oncology, Russian Federation; ^1^Biobank of Research, IRCCS Azienda Ospedaliero-Universitaria di Bologna, 40138 Bologna, Italy; ^2^Department of Medical and Surgical Sciences, Bologna University School of Medicine, 40126 Bologna, Italy; ^3^Reconstructive Orthopaedic Surgery Innovative Techniques-Musculoskeletal Tissue Bank, IRCCS Istituto Ortopedico Rizzoli, 40136 Bologna, Italy; ^4^Department of Hematology, Salus Hospital, 42123 Tirana, Albania

**Keywords:** blastic plasmacytoid dendritic cell neoplasm, microenvironment, targeted therapy, CD123, genomics, epigenetic dysregulation

## Abstract

**Background:**

Blastic plasmacytoid dendritic cell neoplasm (BPDCN) is a rare and aggressive hematologic malignancy originating from precursors of plasmacytoid dendritic cells (pDCs). While normal pDCs are crucial producers of type I interferon (IFN-I) and activators of anti-tumor immunity, BPDCN cells paradoxically lose these functions and create a profoundly immunosuppressive tumor microenvironment (TME).

**Methods:**

This systematic review synthesizes recent genomic and molecular insights to detail the intricate connection between the disease’s intrinsic landscape and its immune evasion mechanisms, with the goal of informing novel therapeutic strategies.

**Results:**

The genomic architecture of BPDCN is marked by frequent cytogenetic abnormalities and pervasive epigenetic dysregulation, defined by recurrent mutations in Tet methylcytosine dioxygenase 2 (*TET2*) and Additional sex combs like 1 (*ASXL1*). Furthermore, the survival of malignant cells is sustained by key oncogenic pathways, including the transcription factor 4 (TCF4)-bromodomain-containing protein 4 (BRD4) network and the high expression of the interleukin-3 receptor alpha chain (CD123). Within the TME, these genomic alterations drive T cell exhaustion, suppress natural killer (NK) cell and macrophage function, and promote the expression of immune checkpoints such as programmed death-ligand 1 (PD-L1), while also establishing an immunosuppressive cytokine milieu rich in interleukin-10 (IL-10) and transforming growth factor-beta (TGF-β). The approval of tagraxofusp, the first CD123-targeted therapy, has revolutionized treatment by achieving high response rates and facilitating allogeneic stem cell transplantation (allo-SCT), which remains the only potentially curative option. However, the frequent development of resistance highlights the need for a new paradigm.

**Discussion:**

Future directions focus on combination therapies that simultaneously address multiple pathways, such as combining CD123-targeted agents with epigenetic modulators or B-cell lymphoma 2 (BCL2) inhibitors like venetoclax to achieve deeper and more durable responses. This integrated approach is essential for advancing precision medicine and improving outcomes for patients facing this challenging neoplasm.

## Graphical abstract

**Graphical abstract.**
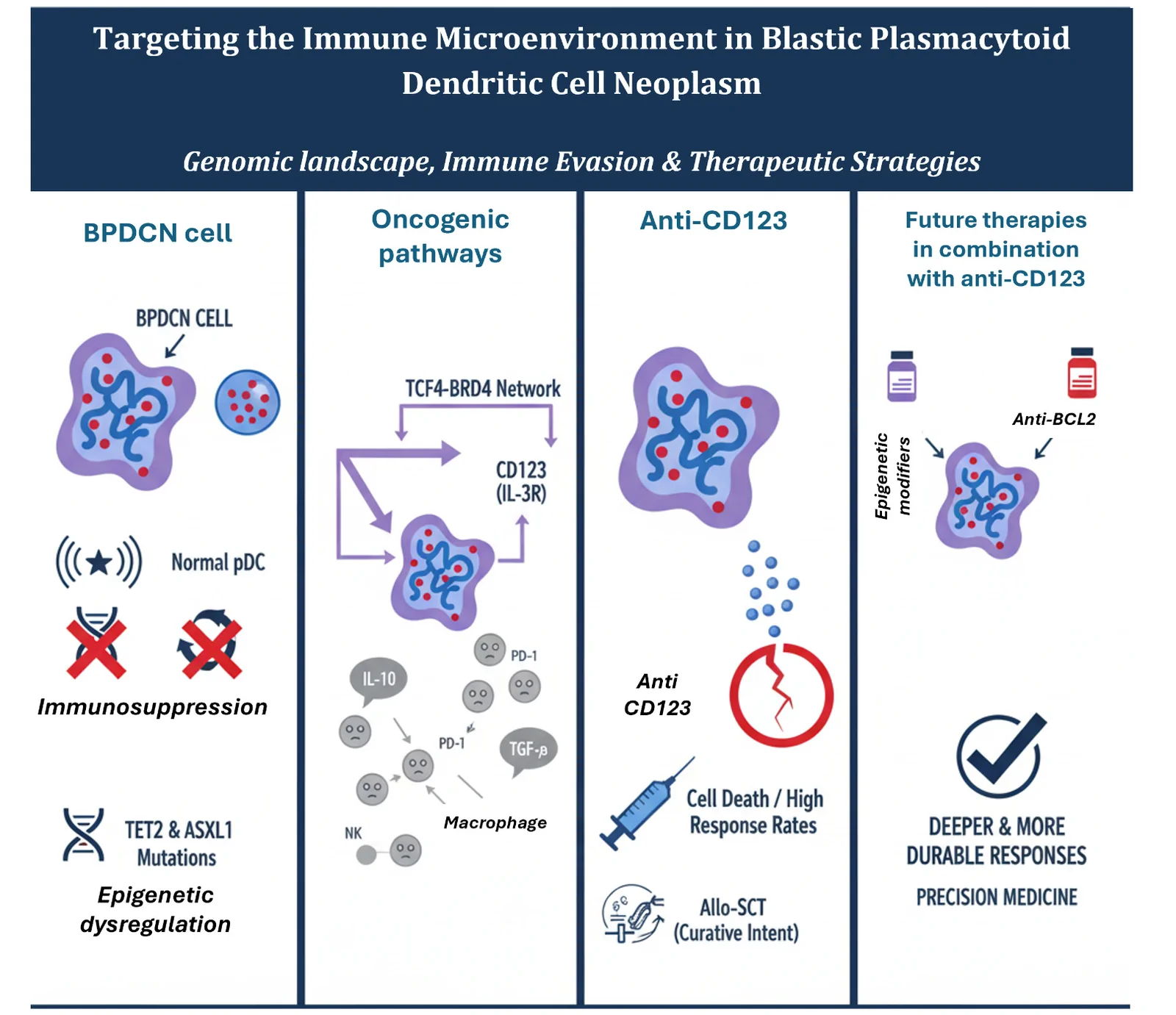
** Interleukin-3 receptor alpha chain (CD123)-directed immunotherapy in blastic plasmacytoid dendritic cell neoplasm (BPDCN): from immune evasion to precision medicine.** allo-SCT: allogeneic stem cell transplantation.

## Introduction

Blastic plasmacytoid dendritic cell neoplasm (BPDCN) is a rare and aggressive hematologic neoplasm, classified by the World Health Organization as a neoplasm derived from precursors of plasmacytoid dendritic cells (pDCs) [1–3]. This clinical entity, although uncommon, presents an extremely serious picture, with a historically poor prognosis and a median survival that, in the absence of targeted therapies, was between 12 and 24 months. The median age at diagnosis is generally advanced, between 60 and 70 years [4], with a marked prevalence in males [4, 5]. The clinical presentation is characteristically heterogeneous, but in the majority of cases (about 90%) it manifests with purplish skin lesions, which can appear as papules, plaques, or nodules [1, 2, 4]. The disease also frequently involves bone marrow, lymph nodes, and other organs [1–3, 6].

BPDCN presents a fascinating biological paradox that guides its pathogenesis and therapeutic challenges. Normal pDCs are crucial components of the innate immune response, known for their exceptional ability to produce massive quantities of type I interferon (IFN-I) in response to pathogenic, particularly viral, stimuli [3, 7]. This makes them central players in coordinating the antiviral and anti-tumor immune response. However, malignant BPDCN cells express distinctive pDC phenotypic markers [such as cluster of differentiation 4 (CD4), CD56, interleukin-3 receptor alpha chain (CD123), transcription factor 4 (TCF4), and T cell leukemia/lymphoma 1 (TCL1)] [3, 8–10]. Almost completely lose their immunogenic function. On the contrary, they are intrinsically inefficient in producing IFN-I and in antigen presentation, as well as creating an actively immunosuppressive microenvironment that favors their own proliferation and escape from host immune surveillance [1].

This manuscript provides a critical synthesis of current advances, integrating insights from a targeted review of the most recent and up-to-date literature. The objective is to explore the deep interconnectedness between the genomic alterations of BPDCN and its immune microenvironment [11, 12].

Unlike previous reviews focusing separately on BPDCN genomics or tumor microenvironment (TME), this work uniquely elucidates how epigenetic dysregulation drives immune evasion.

## Materials and methods

This systematic review synthesizes data from a systematic literature search conducted on PubMed, Scopus, and Web of Science (January 1, 2020–December 31, 2025) using controlled terms: “Blastic Plasmacytoid Dendritic Cell Neoplasm” AND (“genomics” OR “epigenetics” OR “tumor microenvironment” OR “CD123” OR “targeted therapy”). The initial search yielded 287 records. After removing duplicates (*n* = 73) and screening titles and abstracts, 156 articles were assessed for eligibility. Following full-text review, 89 studies met inclusion criteria, comprising: original research articles with ≥ 10 BPDCN patients (*n* = 42 studies, total 1,847 patients), clinical trials registered on ClinicalTrials.gov (*n* = 12 trials, including phase I-II studies), comprehensive genomic analyses (*n* = 18 studies), TME characterization studies (*n* = 11 studies), and relevant high-impact reviews (*n* = 6). Inclusion criteria comprised peer-reviewed studies (*n* ≥ 10 patients), clinical trials (ClinicalTrials.gov), and relevant preprints (medRxiv/bioRxiv). Data were systematically extracted for genomic alterations (type, frequency, biological function), TME mechanisms (immune evasion pathways), and therapeutic outcomes [overall response rate (ORR), resistance]. Critical analysis established causal relationships between epigenetic dysregulation and immunosuppressive TME formation, informing novel combination therapy rationales. This systematic review was designed according to PRISMA statement [13] (Figure 1).

**Figure 1:**
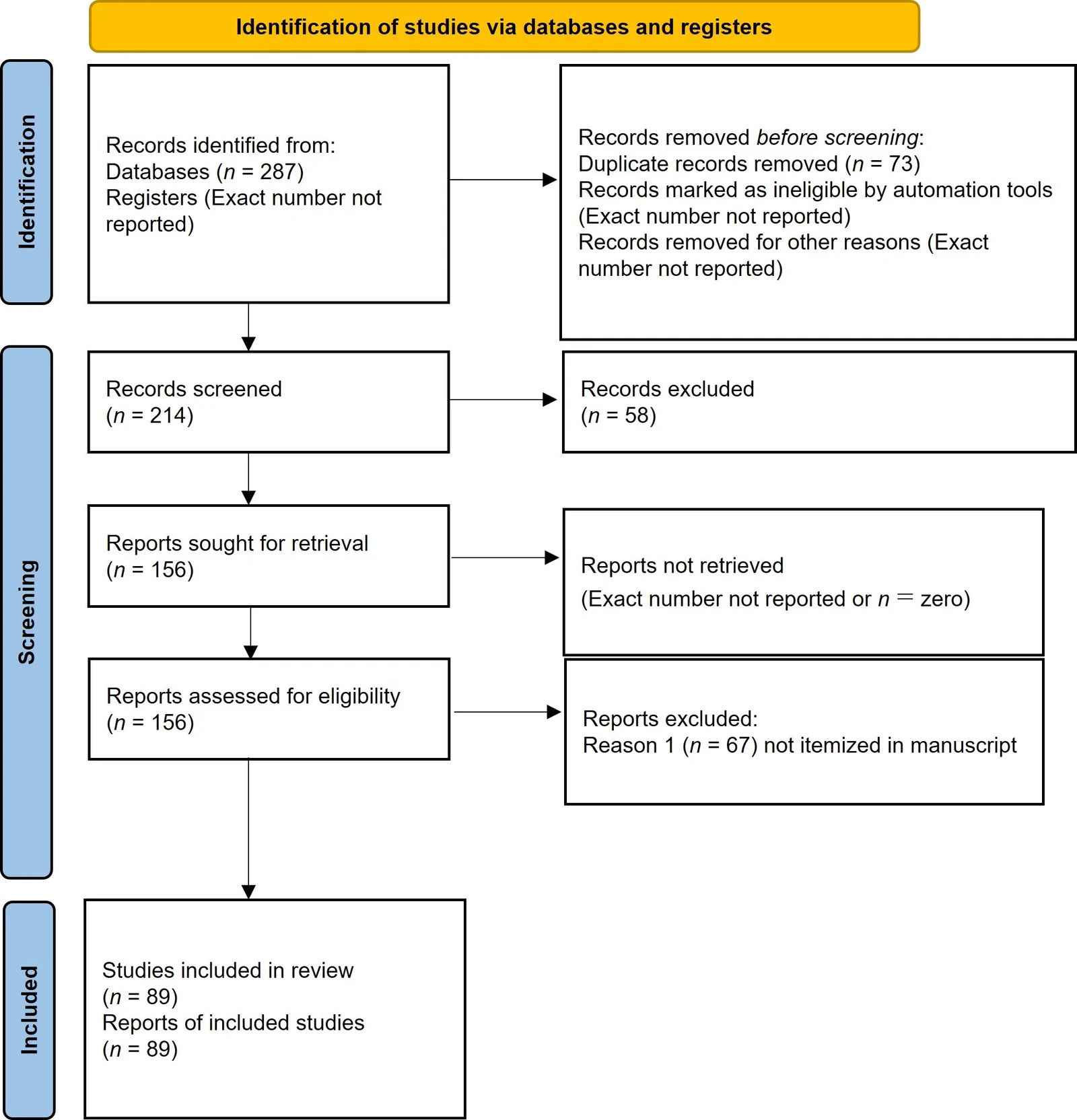
**Study workflow according to the PRISMA 2020 statement.** Adapted from [13]. © Author(s) 2021. CC BY 4.0.

## Results

### Genetic-cytogenetic features and diagnostic role

Cytogenetic abnormalities are a common finding in BPDCN, with about 60% of cases presenting complex karyotypes defined by ≥ 3 aberrations [2]. Recurrent chromosomal deletions include those affecting the long arms of chromosomes 5, 12, and 13, and the short arm of chromosome 9 (9p21) [2]. In particular, the deletion of 9p21.3 has been associated with a worse prognosis [14]. Although these abnormalities and rearrangements of genes like *MYC* and *MYB* are frequently deregulated [15, 16]; however, they do not offer absolute diagnostic specificity, as they are also found in other hematologic neoplasms [15]. For this reason, the definitive diagnosis is based on a combination of histology, cytogenetics, and an accurate immunophenotypic evaluation that identifies the classic triad of markers CD4, CD56, and CD123, in addition to other pDC-specific antigens such as blood dendritic cell antigen 2 (BDCA-2)/CD303, TCF4, and TCL1 [10, 11, 17, 18].

### Epigenetic mutations: the genetic signature of BPDCN

At the molecular level, BPDCN is characterized by a pervasive dysfunction of the epigenetic regulatory program, which represents a distinctive “hallmark” of the disease [1]. Whole-exome sequencing analysis has revealed that most patients had mutations in at least 25 epigenetic modifier genes [1]. The most frequently mutated genes are Tet methylcytosine dioxygenase 2 (*TET2*) and Additional sex combs like 1 (*ASXL1*), found in 57% and 33% of cases, respectively [1].

Mutations in *TET2* and *ASXL1* are often biallelic and appear to be of a founder nature, suggesting that they represent early clonal events in the evolution of the disease [1, 19]. This is not a simple diagnostic marker but a central pathogenetic mechanism. Mutations in these genes not only confer a proliferative advantage to neoplastic cells but also compromise the immune functions typical of pDCs. For example, the loss of function of *TET2*, an enzyme that regulates deoxyribonucleic acid (DNA) demethylation, leads to aberrant methylation that can silence the expression of crucial pro-immunogenic genes for the anti-tumor response [20]. This explains the loss of the ability to produce IFN-I and contributes to creating a microenvironment that cannot be effectively recognized and countered by the host’s immune system [1, 20]. Epigenetic dysfunction, therefore, is an upstream mechanism that provides a direct explanation for the biological paradox of BPDCN.

### Dependence on specific oncogenic signaling pathways

In addition to the epigenetic signature, BPDCN cells show a crucial dependence on specific signaling pathways for their survival. TCF4 has been identified as a “master regulator” of the BPDCN oncogenic program [21]. The oncogenicity of TCF4 is supported by the bromodomain-containing protein 4 (BRD4), which acts in genomic regions called super-enhancers, a molecular architecture that makes the pathway susceptible to inhibition by drugs known as bromodomain and extra-terminal domain inhibitors (BETi) [21].

Another intrinsic vulnerability is the ubiquitous and high-intensity expression of CD123 on the surface of malignant cells [22, 23]. This overexpression, which distinguishes BPDCN cells from normal hematopoietic cells, makes CD123 an ideal therapeutic target, on which the most effective available targeted therapies are based [11, 17, 24].

The advent of advanced omics analysis technologies, such as single-cell ribonucleic acid sequencing (scRNA-Seq), has further refined the understanding of BPDCN [25]. These techniques have revealed that BPDCN is not a single cell-type neoplasm but is composed of heterogeneous cell populations with distinct molecular profiles and roles in disease progression [18, 25]. A recent noteworthy discovery is the identification of new gene fusions, such as *MYB::ZFAT*, which lead to a dysregulated activation of the MYB TCF [25]. This rearrangement contributes to the complexity of the disease and its heterogeneity, highlighting how oncogenic signaling pathways can differ among patients [25]. The integration of this molecular data has allowed for the mapping of detailed genetic and cytogenetic architecture, crucial for diagnosis and for defining personalized treatments (Table 1).

**Table 1:** Recurrent genomic and cytogenetic alterations in blastic plasmacytoid dendritic cell neoplasm (BPDCN).

**Gene/Abnormality**	**Locus**	**Type of alteration**	**Mutation frequency (%)**	**Key biological function**	**References**
Tet methylcytosine dioxygenase 2 (*TET2*)	4q24	Missense, frameshift, nonsense mutation	57	Epigenetic regulation (DNA demethylation)	[1]
Additional sex combs like 1 (*ASXL1*)	20p11	Frameshift, nonsense mutation	33	Epigenetic regulation (histone modification)	[1]
*IDH2* (isocitrate dehydrogenase 2)	15q26.1	Point mutation	Rare, but present	Epigenetic regulation (isocitrate metabolism)	[2]
Transcription factor 4(*TCF4*)	18q21.2	Overexpression	Common	TCF (oncogenic master regulator)	[21]
Interleukin-3 receptor alpha chain (*CD123*) (*IL3RA*)	Xp11.3/Yp11.3	Overexpression	Universally high	Interleukin-3 receptor, therapeutic target	[8]
Chromosome 5q	5q31–q33	Deletion	Recurrent	Unknown, linked to other myeloid neoplasms	[2]
*CDKN2A/B* (cyclin-dependent kinase inhibitor 2A/2B)	9p21	Deletion (p21)	Common	Unknown, associated with worse prognosis	[2]
*MYC*	8q24.21	Rearrangement	Common	TCFs, cell cycle control	[15]
*MYB*	6q23.3	Rearrangement	Common	TCFs, cell cycle control	[15]
*RB1* (retinoblastoma 1)	13q14.2	Loss-of-function alterations: truncating mutations with loss of heterozygosity (LOH) at the RB1 locus, and broader 13q14 deletions	Uncommon	Tumor suppressor controlling Gap 1 to synthesis phase (cell cycle transition) (G1–S) cellcycle transition	[26]
IKZF1 (IKAROS family zinc finger 1)	7p12.2	Predominantly focal interstitial deletions and unbalanced rearrangements at 7p12.2 causing loss of function	Common	TCF essential for plasmacytoid dendritic cell (pDC) precursor differentiation	[27]

### The immune microenvironment of BPDCN: an immuno-tolerant niche

The TME of BPDCN is not merely a passive environment in which malignant cells proliferate undisturbed, but an ecosystem actively and dynamically “sculpted” by neoplastic cells to promote immuno-tolerance and escape from immune surveillance [28] (Figure 2).

**Figure 2:**
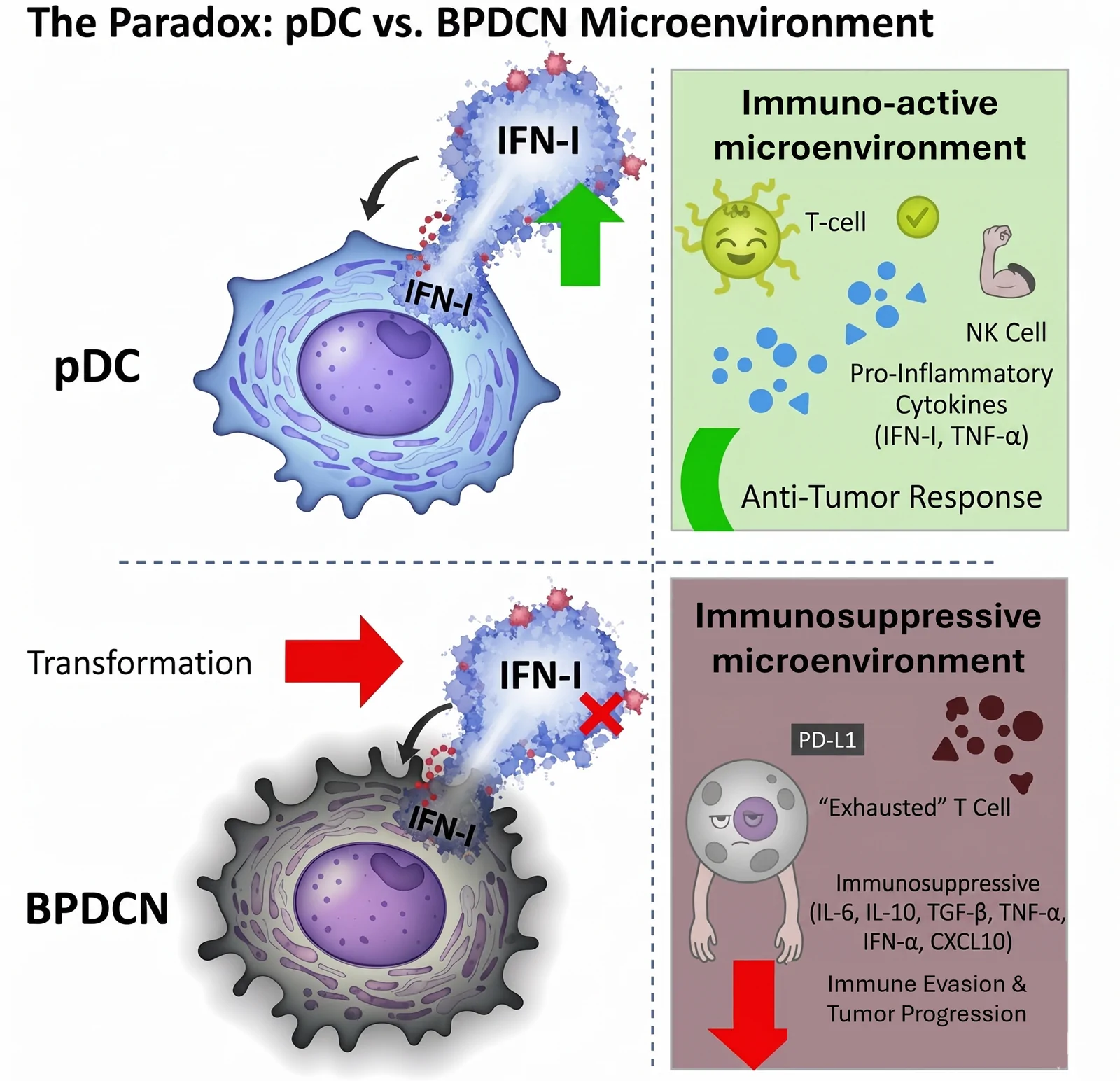
**Comparison of normal plasmacytoid dendritic cells (pDCs) and malignant blastic plasmacytoid dendritic cell neoplasm (BPDCN) cells showing altered type I interferon (IFN-I) signaling.** The BPDCN microenvironment induces T cell exhaustion and natural killer (NK) cell suppression through interleukin-10 (IL-10), transforming growth factor-beta (TGF-β) and programmed death-ligand 1 (PD-L1). Green arrow: intact anti-tumor effect of IFN-I; red cross: abolished anti-tumor function of IFN-I secretion (BPDCN continues to produce IFN-I); red arrow: malignant transformation of pDC to BPDCN. CXCL10: C-X-C motif chemokine ligand 10.

### Immune evasion and immunosuppression mechanisms

Unlike “hot” solid tumors that present with intense infiltration of active immune cells, BPDCN is characterized by a TME with a reduced presence or dysfunction of key cell populations such as T lymphocytes, natural killer (NK) cells, and macrophages [7, 25]. BPDCN cells, although derived from pDCs, lose their immunogenic function and become ineffective in producing IFN-I and in antigen presentation [8, 20]. Instead of triggering an immune response, they contribute to an immunosuppressive environment that ensures their survival.

Studies based on scRNA-Seq have revealed that T lymphocytes present in the BPDCN TME show a state of “exhaustion,” a phenomenon characterized by the loss of cytotoxic ability following chronic antigenic stimulation [25]. In parallel, a reduced functionality of NK cells and macrophages has been observed; although present, they are unable to carry out an effective anti-tumor activity [25]. This cellular dysfunction is the result of complex and synergistic escape mechanisms.

BPDCN cells have developed multi-level strategies to disarm the host’s immune response. First, the expression of immune checkpoint ligands, such as programmed death-ligand 1 (PD-L1), is a crucial mechanism for inducing immune tolerance [22, 29]. Studies have shown that PD-L1 expression is detectable in 47.6% of BPDCN cases and correlates with an increased presence of programmed cell death protein 1 (PD-1)-positive cells within the tumor [30]. The binding between PD-L1 and its receptor PD-1 on T lymphocytes induces a powerful inhibition of their function, leading to their exhaustion and allowing tumor cells to evade immune surveillance [29]. Furthermore, the BPDCN TME is dominated by a cytokine profile that promotes immunosuppression rather than inflammation [31]. Malignant cells and stromal cells recruited into the TME produce immunosuppressive molecules such as interleukin-10 (IL-10) and transforming growth factor-beta (TGF-β) [7, 31]. These cytokines directly inhibit the maturation and function of dendritic cells and effector T cells, further compromising the immune response [32]. Finally, the nuclear factor kappa-light-chain-enhancer of activated B cells (NF-κB) pathway has been identified as a key “driver” in the survival of BPDCN cells and their ability to modulate the TME [12, 33]. Aberrant activation of NF-κB contributes not only to the proliferation of neoplastic cells but also to the production of cytokines and chemokines that ensure the persistence of an immunosuppressive environment [12, 33, 34] (Figure 3).

**Figure 3:**
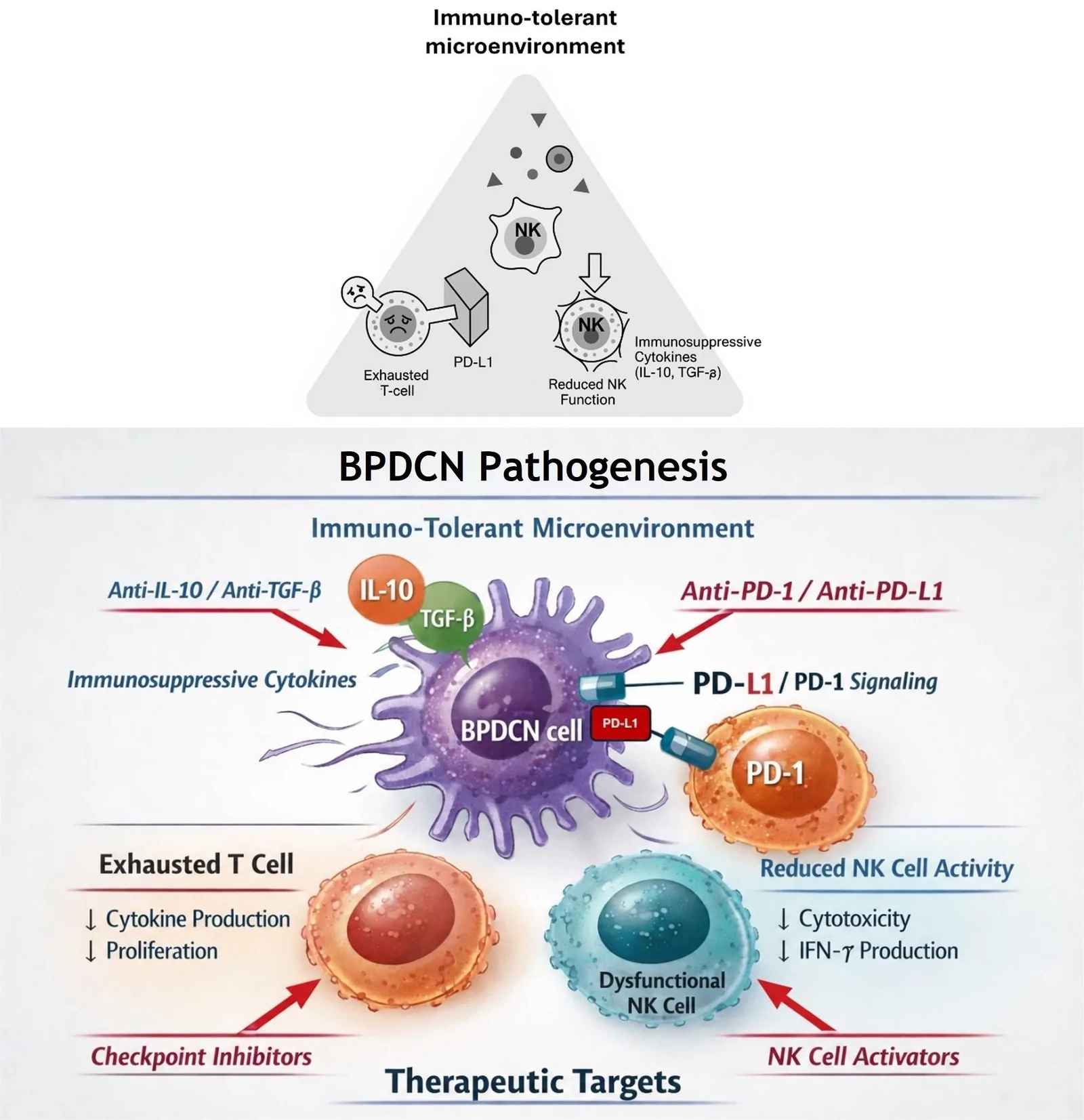
**An immuno-tolerant microenvironment characterized by T cell exhaustion and reduced natural killer (NK) cell activity. Immunosuppressive cytokines [interleukin-10 (IL-10), transforming growth factor-beta (TGF-β)] and programmed death-ligand 1 (PD-L1) signaling promote immune evasion.** BPDCN: Blastic plasmacytoid dendritic cell neoplasm.

### Spatial, functional, and disease-stage heterogeneity of the BPDCN microenvironment

Beyond the general immunosuppressive features of the BPDCN TME, emerging evidence highlights significant spatial, functional, and temporal heterogeneity that may critically influence disease progression and therapeutic response. Recent advances in spatial profiling technologies, including multiplex immunohistochemistry and imaging mass cytometry, have revealed that the BPDCN microenvironment exhibits regional diversity in cellular composition and functional states across different tissue compartments, immune cell distribution patterns differ between peripheral tumor zones, central tumor regions, and interfaces with normal tissue, with distinct immune cell densities and activation states characterizing each spatial domain [35, 36]. This spatial heterogeneity may explain variable responses to immunotherapeutic interventions, as effector immune cells must navigate physically and biochemically distinct microenvironmental niches to exert anti-tumor activity.

Furthermore, the functional state of immune populations within the BPDCN TME evolves dynamically during disease progression and in response to therapy. Single-cell analyses have demonstrated that T cells within BPDCN tissues exhibit a spectrum of exhaustion states rather than a uniform phenotype, with subpopulations retaining proliferative capacity and cytotoxic potential that may be therapeutically exploitable [25]. Similarly, tumor-associated macrophages (TAMs) display functional plasticity, with the capacity to shift between immunosuppressive M2-like and pro-inflammatory M1-like phenotypes depending on local cytokine milieu and metabolic constraints. Disease stage also impacts TME composition: early-stage cutaneous BPDCN lesions may exhibit different immune infiltration patterns compared to advanced systemic disease with bone marrow involvement, potentially reflecting distinct evolutionary trajectories and microenvironmental selection pressures [26].

These insights underscore the need for microenvironmental reprogramming strategies that complement direct tumor-targeted therapies, and several approaches are under preclinical and early clinical investigation. STING (stimulator of interferon genes) and TLR (toll-like receptor) agonists can activate innate immune signaling pathways, inducing IFN-I production, enhancing antigen presentation, and promoting recruitment and activation of effector T cells and NK cells; by restoring the immunogenic functions that normal pDCs possess but BPDCN cells have lost, STING and TLR agonists may theoretically overcome the intrinsic IFN-I deficiency characteristic of BPDCN [7, 37–40]. In parallel, CSF-1R (colony-stimulating factor 1 receptor) inhibitors can deplete or functionally reprogram immunosuppressive TAMs within the TME, reducing IL-10 and TGF-β secretion and facilitating CD8+ T cell infiltration and cytotoxic activity, and preclinical models demonstrate that CSF-1R blockade synergizes with immune checkpoint inhibitors and adoptive T cell therapies by alleviating myeloid-mediated immune suppression [41–43]. Building on these concepts, combination immunomodulatory approaches integrating checkpoint inhibition (anti-PD-1/PD-L1), innate immune activation (STING/TLR agonists), and myeloid reprogramming (CSF-1R inhibitors) represent rational strategies to simultaneously address multiple layers of immune evasion in BPDCN, and such combinations hold promise for converting the “cold” immunosuppressive BPDCN TME into a “hot” inflammatory microenvironment permissive to effective anti-tumor immunity. Collectively, these findings emphasize that the BPDCN TME is not a static or homogeneous entity but rather a complex, spatially organized, and dynamically evolving ecosystem, and therapeutic strategies must therefore account for this heterogeneity and target multiple microenvironmental components to achieve durable disease control; future research integrating spatial transcriptomics, longitudinal TME profiling across disease stages, and rational immunomodulatory combinations will be essential for advancing precision medicine approaches in BPDCN.

The link between genomic alterations and immune evasion mechanisms is profound. Mutations in key genes like *TET2* and *ASXL1* not only compromise the immunogenic functions of malignant pDCs but also predispose to the activation of pathways that promote immunosuppression. In this context, the expression of PD-L1 and the production of IL-10 are not random events, but the result of a genomic program that has “reprogrammed” BPDCN cells from sentinels of the immune system to facilitators of immune evasion. This cascading mechanism explains the extreme aggressiveness of the disease and justifies the therapeutic approach that aims to restore immune surveillance [7, 44].

The in-depth understanding of the genomic profile and immune microenvironment of BPDCN has allowed for a shift from non-specific chemotherapeutic regimens to targeted therapies that exploit the intrinsic vulnerabilities of neoplastic cells (Table 2) [17].

**Table 2:** Overview of Targeted Therapies for blastic plasmacytoid dendritic cell neoplasm (BPDCN).

**Therapeutic agent**	**Mechanism of action**	**Molecular target**	**Key clinical results**	**Main toxicities**	**Development status**	**Reference**
Tagraxofusp	Recombinant fusion protein [interleukin-3 (IL-3) + diphtheria toxin]	Interleukin-3 receptor alpha chain (CD123)	Overall response rate (ORR) 75–90% in treatment-naive; bridge to allogeneic stem cell transplantation (allo-SCT).	Capillary leak syndrome (CLS), hepatotoxicity.	Approved (US, EU)	[4]
Pivekimab sunirine (IMGN632)	Antibody-drug conjugate (ADC)	CD123	ORR 21% [acute myeloid leukemia (AML)], promising preliminary activity in BPDCN.	Fever, neutropenia, peripheral edema, veno-occlusive disease.	In clinical trials (Breakthrough Therapy Designation)	[45]
Venetoclax	B-cell lymphoma 2 (BCL2) inhibitor	BCL2	Significant responses in relapsed/refractory (R/R) patients; in clinical trials in combination.	Cytopenias, tumor lysis syndrome.	In clinical trials (for BPDCN)	[46]
Azacitidine	Hypomethylating agent	Epigenetic regulators	Remodels tumor microenvironment (TME), reactivates pro-immunogenic genes.	Cytopenias, gastrointestinal toxicity.	In clinical trials in combination	[24]

### Targeting CD123: the era of targeted therapies

The universally high expression of CD123 has made this receptor the main target for targeted therapies. Tagraxofusp (also known as Elzonris) is the first and only therapy specifically approved for BPDCN [10, 23, 47]. Its mechanism of action is based on a recombinant fusion protein that combines IL-3 (the CD123 ligand) with a truncated diphtheria toxin [10, 24, 48]. This structure allows for the selective release of the cytotoxic payload into cells that overexpress CD123, inhibiting protein synthesis and inducing cell death. The results of a phase II trial, the largest prospective study on BPDCN, have shown an unprecedented efficacy [49]. The drug induced an ORR of 75% in treatment-naive patients, with 57% of them achieving a complete response [CR/clinical CR(CRc)] [47–49]. Interestingly, tagraxofusp has proven to be particularly effective as a “bridge” therapy for allogeneic stem cell transplantation (allo-SCT), which remains the only potentially curative option for eligible patients [10, 48].

However, the use of Tagraxofusp is not without challenges. The main reported toxicity was capillary leak syndrome (CLS), reported in 21% of patients, which can be severe but can be improved with careful patient selection (based on albumin levels and cardiac function) and intensive monitoring, especially during the first treatment cycle [4, 48, 50, 51].

### New immunotargeting agents

Pivekimab sunirine (IMGN632) is a next-generation antibody-drug conjugate (ADC) designed to selectively target CD123, which is uniformly and highly expressed on BPDCN cells. The molecule consists of a high-affinity humanized anti-CD123 monoclonal antibody conjugated via a cleavable linker to a potent DNA-alkylating payload from the indolinobenzodiazepine pseudodimer (IGN) class, enabling efficient intracellular delivery and cytotoxicity at sub-picomolar concentrations [45]. Compared with first-generation CD123-directed therapies such as tagraxofusp, IMGN632 demonstrates improved target selectivity and a more favorable therapeutic index. Notably, across early-phase clinical trials, IMGN632 has not been associated with cases of CLS, a major dose-limiting toxicity observed with tagraxofusp, thereby offering a more manageable and predictable safety profile [45].

Preclinical studies have shown robust antitumor activity of IMGN632 in BPDCN xenograft models, including those with low antigen density, and early clinical data indicate meaningful single-agent activity in both treatment-naïve and relapsed/refractory BPDCN patients [45]. Responses have been observed even in heavily pretreated individuals, supporting its potential role across different lines of therapy and as a backbone for combination strategies.

Beyond ADC-based approaches, several additional CD123-directed immunotherapies are currently in development. These include bispecific antibodies that simultaneously engage CD123 on tumor cells and CD3 or CD16 on effector T cells or NK cells, thereby redirecting cytotoxic immune responses toward BPDCN blasts. In parallel, CD123-targeted chimeric antigen receptor (CAR) T cell therapies have demonstrated potent preclinical efficacy and are entering early-phase clinical evaluation. Collectively, these emerging immunotargeting strategies underscore the central role of CD123 as a therapeutic vulnerability in BPDCN and highlight the rapid evolution of immune-based treatments aimed at improving outcomes while minimizing treatment-related toxicity [6, 10, 11, 51].

### The role of epigenetic and other targeted therapies

Genomic discoveries have paved the way for therapies that go beyond just targeting CD123. Mutations in *TET2* and *ASXL1*, together with gene expression profiling studies, have provided the biological rationale for the use of hypomethylating agents such as azacitidine and decitabine [1]. These agents exert not only direct cytotoxic effects on malignant cells but also promote epigenetic reprogramming of the TME by restoring the expression of pro-immunogenic genes that are frequently silenced in BPDCN, including components of the antigen-presentation machinery [human leukocyte antigen (HLA) class I and II genes, beta-2-microglobulin (*B2M*)], interferon-stimulated genes [interferon-induced protein with tetratricopeptide repeats 1 (*IFIT1*), *IFIT3*, *MX1*], T helper 1 (Th1)-polarizing cytokines and chemokines [C-X-C motif chemokine ligand 9 (*CXCL9*), *CXCL10*, *CXCL11*], and costimulatory or immune-regulatory molecules [*CD40*, intercellular adhesion molecule 1 (*ICAM1*)]. This immunologic reactivation may enhance tumor immunogenicity, facilitate immune cell recruitment, and sensitize BPDCN cells to immune-based therapies [52].

In addition, it has been shown that BPDCN is uniformly dependent on the anti-apoptotic protein B-cell lymphoma 2 (BCL2), which allows malignant cells to evade programmed cell death signals [46]. This vulnerability makes BPDCN highly sensitive to drugs like Venetoclax, a powerful BCL2 inhibitor [1, 2]. In parallel, BRD4 inhibitors (BETi) are able to disrupt the TCF4-BRD4 signaling pathway, another crucial axis for the survival of neoplastic cells [21].

Of note, the mechanism responsible for BCL2 deregulation has not been defined yet.

### The need for combination therapies to overcome resistance

Despite the initial effectiveness of tagraxofusp, resistance to therapy often develops, sometimes through mechanisms related to the epigenetic silencing of genes that encode for the diphtheria toxin’s targets [25, 53]. Understanding these resistance pathways has led to a paradigm shift towards combination therapies. The biological rationale is to simultaneously target multiple intrinsic vulnerabilities of BPDCN cells: dependence on CD123, epigenetic dysregulation, and the anti-apoptotic pathway [25, 48, 49, 54]. Ongoing clinical trials are evaluating the combination of tagraxofusp with hypomethylating agents like azacitidine and/or with BCL2 inhibitors like venetoclax [17]. This integrated strategy promises to overcome resistance mechanisms and achieve deeper, more durable responses, reducing the likelihood of relapse [24]. (Figure 3). The shift from a monoclonal approach to a strategy that recognizes a network of interconnected vulnerabilities reflects the evolution of precision medicine for BPDCN [18, 47] (Figure 4; Table 2).

**Figure 4:**
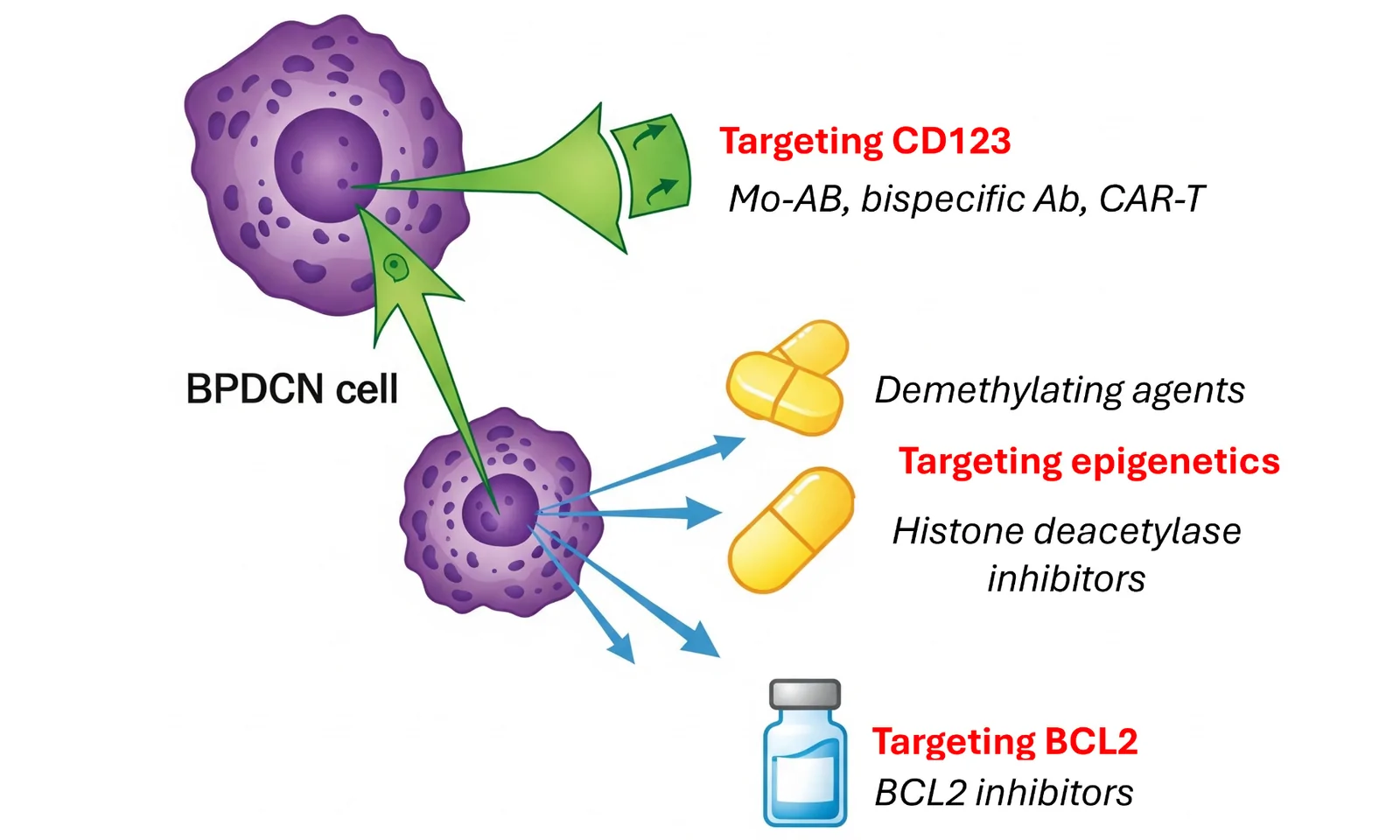
**Therapeutic strategies targeting the blastic plasmacytoid dendritic cell neoplasm (BPDCN) microenvironment include interleukin-3 receptor alpha chain (CD123) inhibition, epigenetic modulators, and B-cell lymphoma 2 (BCL2) blockade.** These approaches exploit tumor dependencies to enhance immune-mediated killing. CAR: chimeric antigen receptor.

## Discussion

The understanding of BPDCN has evolved from a rare nosological entity to a disease with a well-defined molecular architecture, which is at the heart of its biology and aggressiveness. The profound interconnectedness between genomic alterations (particularly epigenetic dysfunction) and the formation of an immunosuppressive microenvironment has been established as the key factor determining clinical behavior and therapeutic response [17].

Despite the revolutionary success of tagraxofusp, which has transformed the therapeutic landscape, the management of BPDCN remains a significant challenge. The main challenges include managing toxicity, understanding long-term resistance mechanisms, and the lack of reliable predictive biomarkers to guide therapy selection [47, 55].

The prospects for future research are increasingly centered on precision medicine approaches and rational combination therapies [9]. The systematic application of high-resolution techniques such as scRNA-seq will enable an even more granular dissection of the TME, facilitating the identification of rare immune and stromal cell populations that may drive immune escape, therapeutic resistance, or clinical prognosis [25]. In parallel, the development of novel agents with improved efficacy and toxicity profiles, including IMGN632 and other next-generation ADCs, is expected to further expand the therapeutic armamentarium for BPDCN [56]. Importantly, increasing attention is being directed toward microenvironmental reprogramming strategies aimed at restoring effective local antitumor immunity. These include inhibitors of the CSF-1R, which can deplete or re-educate immunosuppressive TAMs, as well as innate immune agonists targeting the STING and TLR pathways, capable of inducing IFN-I signaling, enhancing antigen presentation, and promoting T-cell and NK cell recruitment. Together, these approaches hold promise for synergizing with CD123-directed therapies and immune-based strategies, ultimately overcoming immune tolerance and improving durable disease control in BPDCN.

The future of BPDCN treatment does not lie in a single “magic bullet” but in an integrated approach that rationally and sequentially combines targeted therapies with the consolidation of allo-SCT [23]. Clinical trials combining agents that target distinct vulnerabilities (CD123, BCL2, epigenetic dysregulation) represent the most promising direction. This therapeutic model aims to overcome resistance, maximize the depth of response, and optimize the use of transplantation, thus offering patients with this aggressive neoplasm the best chance for a lasting remission.

## Abbreviations

ADC: antibody-drug conjugate
allo-SCT: allogeneic stem cell transplantation
AML: acute myeloid leukemia
*ASXL1*: Additional sex combs like 1
*B2M*: beta-2-microglobulin
BCL2: B-cell lymphoma 2
BDCA-2: blood dendritic cell antigen 2
BETi: bromodomain and extra-terminal domain inhibitors
BPDCN: Blastic plasmacytoid dendritic cell neoplasm
BRD4: bromodomain-containing protein 4
CAR: chimeric antigen receptor
CD123: interleukin-3 receptor alpha chain
CD4: cluster of differentiation 4
CDKN2A/B: cyclin-dependent kinase inhibitor 2A/2B
CLS: capillary leak syndrome
CR: complete response
CRc: clinical complete response
CSF-1R: colony-stimulating factor 1 receptor
*CXCL9*: C-X-C motif chemokine ligand 9
DC: dendritic cell
DNA: deoxyribonucleic acid
HLA: human leukocyte antigen
*ICAM1*: intercellular adhesion molecule 1
*IDH2*: isocitrate dehydrogenase 2
*IFIT1*: interferon-induced protein with tetratricopeptide repeats 1
IFN-I: type I interferon
IGN: indolinobenzodiazepine pseudodimer
IKZF1: IKAROS family zinc finger 1
IL-10: interleukin-10
IMGN632: pivekimab sunirine
LOH: loss of heterozygosity
NF-κB: nuclear factor kappa-light-chain-enhancer of activated B cells
NK: natural killer
ORR: overall response rate
PD-1: programmed cell death protein 1
pDCs: plasmacytoid dendritic cells
PD-L1: programmed death-ligand 1
R/R: relapsed/refractory
RB1: retinoblastoma 1
RNA: ribonucleic acid
scRNA-seq: single-cell ribonucleic acid sequencing
STING: stimulator of interferon genes
TAMs: tumor-associated macrophages
TCF4: transcription factor 4
TCL1: T cell leukemia/lymphoma 1
*TET2*: Tet methylcytosine dioxygenase 2
TGF-β: transforming growth factor-beta
Th1: T helper 1
TLR: toll-like receptor
TME: tumor microenvironment

## Declarations

### Availability of data and materials

The datasets supporting the findings of this study are available from the corresponding author upon reasonable request.

### Copyright

© The Author(s) 2026.
